# Genetic characterization of a Coxsackie A9 virus associated with aseptic meningitis in Alberta, Canada in 2010

**DOI:** 10.1186/1743-422X-10-93

**Published:** 2013-03-22

**Authors:** Kanti Pabbaraju, Sallene Wong, Eve N Y Chan, Raymond Tellier

**Affiliations:** 1 Provincial Laboratory for Public Health, 3030 Hospital Drive, Calgary, Alberta, T2N 4W4, Canada; 2 Department of Microbiology, Immunology and Infectious Diseases, University of Calgary, Alberta, Canada; 3 Current address: Department of Speech Pathology and Audiology, University of Alberta, Edmonton, Alberta, Canada

**Keywords:** Human enteroviruses, Coxsackie A9, Aseptic meningitis, Serotyping

## Abstract

**Background:**

An unusually high incidence of aseptic meningitis caused by enteroviruses was noted in Alberta, Canada between March and October 2010. Sequence based typing was performed on the enterovirus positive samples to gain a better understanding of the molecular characteristics of the Coxsackie A9 (CVA-9) strain responsible for most cases in this outbreak.

**Methods:**

Molecular typing was performed by amplification and sequencing of the VP2 region. The genomic sequence of one of the 2010 outbreak isolates was compared to a CVA-9 isolate from 2003 and the prototype sequence to study genetic drift and recombination.

**Results:**

Of the 4323 samples tested, 213 were positive for enteroviruses (4.93%). The majority of the positives were detected in CSF samples (n = 157, 73.71%) and 81.94% of the sequenced isolates were typed as CVA-9. The sequenced CVA-9 positives were predominantly (94.16%) detected in patients ranging in age from 15 to 29 years and the peak months for detection were between March and October. Full genome sequence comparisons revealed that the CVA-9 viruses isolated in Alberta in 2003 and 2010 were highly homologous to the prototype CVA-9 in the structural VP1, VP2 and VP3 regions but divergent in the VP4, non-structural and non-coding regions.

**Conclusion:**

The increase in cases of aseptic meningitis was associated with enterovirus CVA-9. Sequence divergence between the prototype strain of CVA-9 and the Alberta isolates suggests genetic drifting and/or recombination events, however the sequence was conserved in the antigenic regions determined by the VP1, VP2 and VP3 genes. These results suggest that the increase in CVA-9 cases likely did not result from the emergence of a radically different immune escape mutant.

## Background

The enterovirus genome is comprised of a single open reading frame flanked by the 5’ and 3’ untranslated regions (UTRs), and the encoded polyprotein is cleaved to produce the structural and nonstructural proteins [1]. Although most infections are asymptomatic, non-polio enteroviruses are the most common infectious cause of aseptic meningitis [1]. Several outbreaks resulting from different enterovirus serotypes have been described including but not restricted to Coxsackievirus A9 (CVA-9) [2], EV71 [3,4], EV68 [5], Coxsackievirus A24 [6], echovirus 18 [7], echovirus 30 [8,9], and Coxsackievirus A16 [10].

Non-polio enteroviruses were traditionally classified into serotypes, based on a neutralization assay and included 64 classical isolates consisting of Coxsackieviruses A, Coxsackieviruses B, echoviruses and several numbered enteroviruses [11]. Genetic studies led to a new classification scheme, grouping the enteroviruses into species A, B, C and D, although serotype determination is still widely used, including for epidemiological purposes. Typing methods based on sequencing have been previously described in the VP1 [12-14], VP2 [15,16], and VP4 [17] regions. Molecular typing methods based on the P1 genomic region sequences, have been found to yield equivalent results to neutralizing assays, which they have essentially replaced [1,14]. Molecular typing has also led to the characterization of new enterovirus serotypes [4,18-20] and provides information on recombination between enteroviruses. This is a common phenomenon which almost always occurs between viruses belonging to the same species [1,21,22]. Serotype determination remains important for molecular epidemiology, in part because the capsid contains the neutralization epitopes, and a rise or fall in incidence of a serotype can be linked to serotype-specific humoral immunity within the population [21]. The capsid also determines the cellular receptors used by the virus [23] and is thus an important determinant of viral pathogenesis, although non-structural proteins also contribute to the pathogenesis of the virus [24].

An unusually high incidence of aseptic meningitis was noted in Alberta, Canada between March and October 2010 [25]. A high proportion of CSF samples submitted to the Provincial Laboratory For Public Health of Alberta were positive for enteroviruses and serotyping by molecular methods revealed that majority of these enteroviruses belonged to the CVA-9 serotype. The primary goal of this study was to provide a full genetic characterization of the CVA-9 isolates responsible for this outbreak. Secondary goals were to comment on the methods of molecular serotyping for the diagnostic laboratory and on the use of long RT-PCR as a convenient method to obtain the near full-length genomic sequence of enteroviruses, especially when recombination events are involved in the genesis of the viral genome. The genome sequence of these isolates was determined and compared to the sequence of a CVA-9 isolate from Alberta in 2003 and to the prototype CVA-9 sequence (strain: Griggs; D00627) [26]. Serotyping by sequencing within the VP2 region was found to be more reliable than within the VP4 region. A comparison of the different genes revealed a higher nucleotide and amino acid conservation in the structural regions, and analysis of the sequence of the non-structural region pointed to recombination events in the genesis of the 2010 isolate.

## Results

### Detection and serotyping

Of the 4323 samples tested, 213 were positive for enteroviruses (4.93%). The majority of the positives were CSF samples (n = 157, 73.71%) followed by specimens of respiratory origin (n = 17, 7.98%), stools (n = 10, 4.70%) and plasma (n = 8, 3.76%). The rest of this analysis concentrates on CSF samples. A total of 72 positive CSF samples were randomly selected to represent different age groups, geographic locations and disease severity for serotype determination based on phylogenetic clustering of the partial sequence of the VP2 region with the prototype sequences. More than 85% of samples could be typed without the need for culture or nested PCR (data not shown). This RT-PCR detects both enteroviruses and rhinoviruses, and allows for differentiation based on the length of the amplicon.

**Figure 1:**
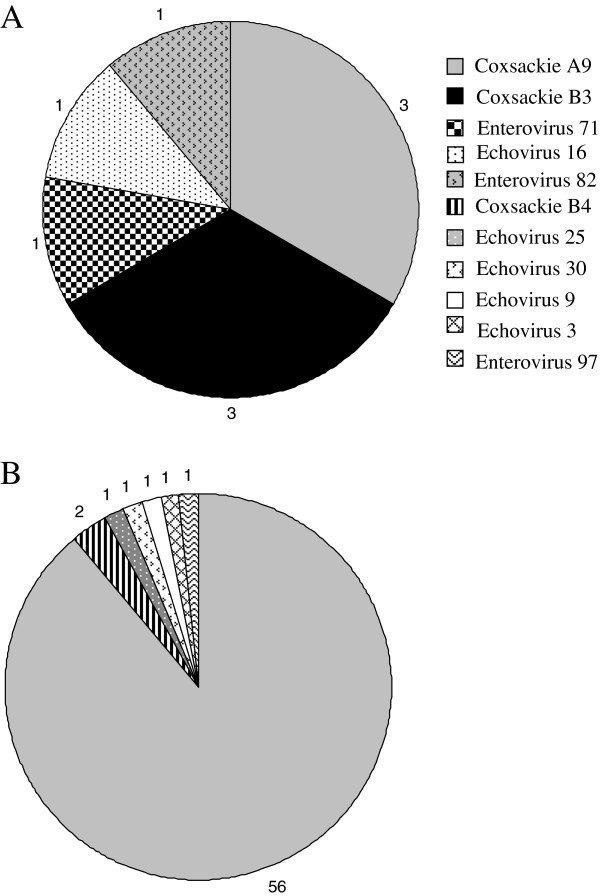
**Distribution of the different serotypes detected in the sequenced samples. A** indicates serotype distribution in samples from patients less than one year and **B** from patients over one year old.

**Figure 2:**
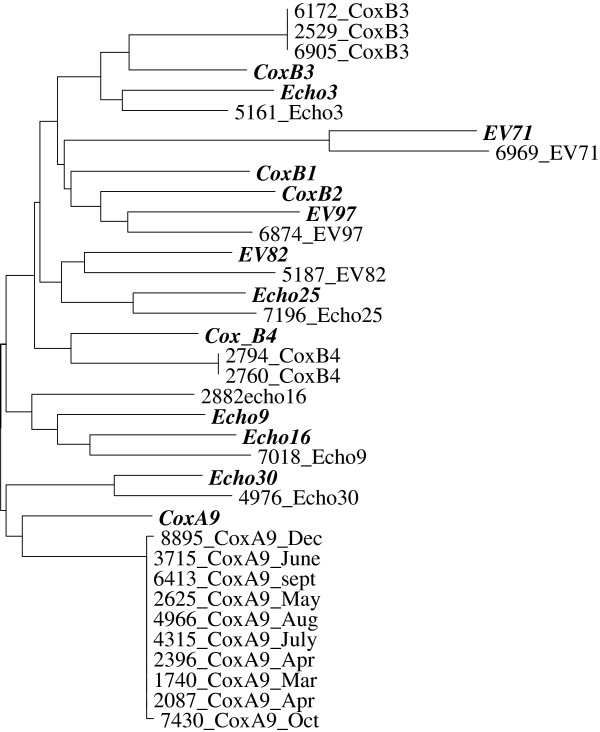
**Phylogenetic tree including representatives of the different serotypes detected and corresponding prototype sequences *(in italics)*.** The alignment used for the phylogenetic tree included 173 bases of the VP2 region from the clinical specimens, the same region from the prototype strains is also included. The Genbank accession number for the prototype strains is included in legend for Table 2. Patient samples are identified using a laboratory number followed by the enterovirus type.

The most common serotype detected was CVA-9 (n = 59, 81.94%) and it was clearly the predominant serotype in the population. Closer examination of age distribution revealed that the majority (n = 56, 94.92%) of the CVA-9 positives were from patients ranging in age from 15 to 29 years, only three (5.08%) CVA-9 positives were detected in children less than a year old. The other frequently detected serotypes were CVB-3 (n = 3) and CVB-4 (n = 2). Figure 1A and 1B show the distribution of the different serotypes detected from infants less than one year old and from patients older than one year. A phylogenetic tree including representatives of the different enterovirus serotypes detected and some representative prototype strains in the VP2 region is indicated in Figure 2. The VP2 sequence from all the sequenced isolates was identical, showing that the outbreak was associated with a clonal or nearly clonal CVA-9 population.

### Age and gender distribution

**Figure 3:**
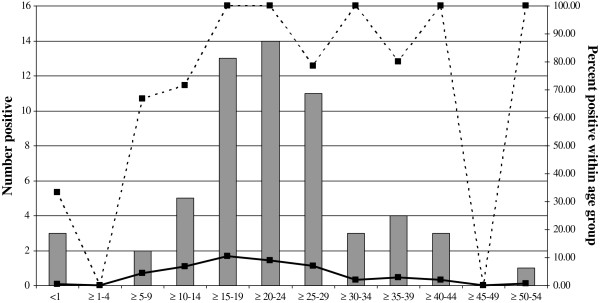
**Age distribution of patients positive for enterovirus CVA-9.** The bars represent the total number of samples positive for CVA-9 and the broken line indicates the percentage of samples positive for CVA-9 among the serotyped samples in each age group. The solid line indicates the percentage of samples positive for all enteroviruses in each age group based on the number of CSF samples tested within that age group.

Of the 59 samples positive for CVA-9, roughly equal numbers were detected in males (n = 30) and females (n = 29). Figure 3 shows the age distribution and percentage of CSF samples positive for CVA-9 within the different age groups tested. The data indicates that the majority of the CVA-9 positives were detected in patients between 15 to 30 years old.

### Seasonality

Samples from January 2010 to December 2010 were used in this study; peak months for the detection of CVA-9 positives in CSF samples were between March and October.

### Comparison of near full length genomes

The genomic sequences of the CVA-9_2003 and CVA-9_2010 have been deposited in GenBank under accession number JQ837913 and JQ837914, respectively. Comparison of the nucleotide and amino acid sequences in each of the gene segments from CVA-9_2003 and CVA-9_2010 to the prototype CVA-9 sequence are shown in Table 1. Nucleotide identity in the 5’ untranslated region (5’ UTR) between CVA-9_2003 and CVA-9_2010 was higher (96.5%) than with the prototype CVA-9 sequence which was around 85%. The nucleotide and amino acid identity was high (around 95%) between CVA-9_2003 and CVA-9_2010 samples in the structural genes (VP4, VP2, VP3 and VP1). The nucleotide identity in the structural genes for both these samples was lower (around 80%) when compared to the prototype CVA-9 sequence but amino acid identity was high (around 90%) indicating that the nucleotide changes consisted mostly of synonymous mutations. CVA-9_2010 showed some sequence diversity from the CVA-9_2003 isolate in the 3’ non-structural domain. This domain encodes the 2A protease, 2B protein, 2C helicase, 3A protein, 3B protein (VPg), 3C protease and 3D protein (polymerase). The nucleotide sequence identity in the 2A protease and 2B protein regions was 96.15% and 93.60% respectively; identity in the 2C helicase, 3A protein and 3D protein was around 80%. The lowest nucleotide identity was observed in the 3B protein at 74.63% and 3C protease at 77.78%, however amino acid conservation in these genes was higher as indicated in Table 1. Comparison of these gene segments to the prototype CVA-9 strain showed a higher percent of amino acid identity as compared to nucleotide identity.

**Table 1:** Percent identity comparison of nucleotide and amino acid sequences for isolates from 2003 (JQ837913), 2010 (JQ837914) and prototype CVA-9 sequence (D00627)

	**2003 Vs 2010**		**2003 Vs Prototype CVA-9**		**2010 Vs Prototype CVA-9**	
**Gene fragment**	**Nucleotide**	**Amino acid**	**Nucleotide**	**Amino acid**	**Nucleotide**	**Amino acid**
5’NCR	96.5	N/A	84.93	N/A	85.5	N/A
VP4	92.65	94.29	79.9	94.12	77.94	94.12
VP2	94.76	94.03	80.95	88.85	80.95	89.23
VP3	95.24	97.51	81.37	98.74	81.65	98.32
VP1	94.92	95.47	82	93.38	81.24	92.05
2A Protease	96.15	97.31	77.78	93.88	79.14	95.24
2B Protein	93.6	83.33	79.46	91.92	80.47	96.97
2C Helicase	82.19	92.96	82.07	97.26	80.87	96.35
3A protein	81.34	93.48	73.03	91.01	74.63	89.89
3B Protein (VPg)	74.63	86.36	79.1	90.91	70.15	86.36
3C Protease	77.78	91.62	81.75	97.27	79.02	95.63
3D Protein (Pol)	80.19	92.09	76.81	96.32	73.69	95.89

**Figure 4:**
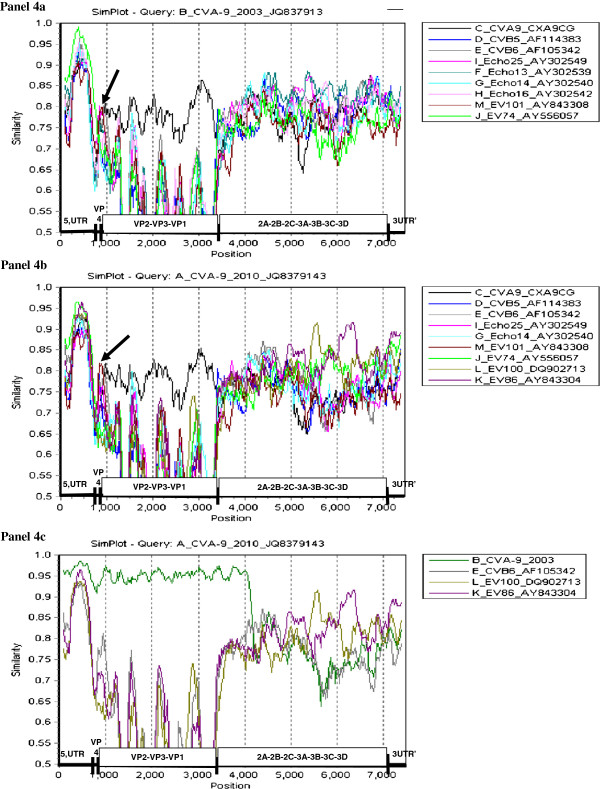
**Simplot analysis of full genome sequences of CVA-9_2003, CVA-9_2010 and related species B sequences.** Genomic regions are indicated at the bottom of each panel based on numbering of the prototype CVA-9 (D00627) 5’UTR:1–722; VP4:723–929; VP2:930–1712; VP3:1713–2426; VP1:2427–3332; 2A:3333–3773; 2B:3774–4069; 2C:4070–5057; 3A:5058–5323; 3B:5324–5390; 3C:5391–5938; 3D:5939–7324; 3’UTR:7325 - >7403. The query sequences used are CVA-9_2003 in panel 4**A** and CVA-9_2010 in panels 4**B** and 4**C**. Arrows in panels 4**A** and 4**B** indicate recombination with EV-101 in the VP4 region.

Phylogenetic analysis comparing the nucleotide sequences of all the species B prototype sequences to CVA-9_2003 and CVA-9_2010 shows that in the VP2 region, these sequences cluster with the prototype CVA-9 sequence with a high bootstrap value; a similar pattern was observed for the VP1 and VP3 regions. However, in the VP4 region, the closest neighbour to CVA-9_2003 and CVA-9_2010 sequences is enterovirus 101 (EV-101) and there is significant divergence from the prototype CVA-9 sequence at the nucleotide level (data not shown). Table 2 lists the nearest neighbour based on phylogenetic analysis for the structural and non-structural gene segments, compared to the gene sequences from prototype strains. As indicated in the table the regions 2A, 2B, and 3B from the CVA-9_2003 and CVA-9_2010 cluster together, however, the 2C, 3A, 3C and 3D regions from CVA-9_2003 and CVA-9_2010 cluster with different enterovirus species B prototype sequences indicating base pair changes or recombination in these regions. The probable mosaic nature of the genome of CVA-9_2003 and CVA-9_2010 was further explored by performing Simplot analysis. Figure 4A compares the sequence of CVA-9_2003 to that of the enteroviruses identified in Table 2 as the most homologous in each gene. All the viruses are highly homologous within the 5’NC, and the higher score of EV-74 may or may not reflect the origin of the 5’UTR segment of the Alberta isolates; nevertheless, sequencing the 5’UTR would not allow for accurate serotyping. It shows that in the VP2-VP3-VP1 region the CVA-9_2003 is most homologous to the prototype CVA-9; it is speculated that the differences are attributable to genetic drifting over the years. It is interesting to note that in the VP4 region the highest homology is with EV-101, suggesting that recombination with EV-101 (or a very similar virus) might have occurred. This suggests that relying only on the VP4 sequence would not allow for accurate serotyping. Simplot analysis also shows that in the non-structural coding region, recombination with other viruses of species B has occurred, although the dominance of individual viruses is not clear enough to attribute the origin of the segments with certainty. Similarly, Figure 4B compares the sequence of CVA-9_2010 to the enteroviruses identified in Table 2. Similar comments can be made regarding the sequence of CVA-9_2010, although in the non-structural domain, identifying CVB-6, EV-86 and EV-100 as contributing to the recombination events appears more convincing. Tables 1 and 2 show that there is a high nucleotide identity between CVA-9_2003 and CVA-9_2010 in the region upstream of that coding for protein 2B. It can be speculated that CVA-9_2010 arose from recombination events between CVA-9_2003, CVB-6, EV-86 and EV-100. This hypothesis is examined by Simplot analysis in Figure 4C. This analysis suggests that CVA-9 (2010 and 2003) are highly homologous from the 5’NC to the 2B domain; recombination with other viruses clearly occurred beyond this point, with contributions from EV-86 and EV-100, and likely from CVB-6.

**Table 2:** Closest neighbour to the 2003 and 2010 samples based on phylogenetic analysis

**Gene fragment**	**Closest neighbour to 2003**	**Closest neighbour to 2010**
5’NCR	EV74	EV74
VP4	EV101	EV101
VP2	CVA-9	CVA-9
VP3	CVA-9	CVA-9
VP1	CVA-9	CVA-9
2A Protease	Echo 25	Echo 25
2B Protein	CVB-5 and echo14	CVB-5 and echo14
2C Helicase	Echo 25	CVB-6
3A protein	Echo 16 and CVB-6	EV 86
3B Protein (VPg)	Echo 14 and CVB-5	Echo 14 and CVB-5
3C Protease	Echo 13	EV 100
3D Protein (Pol)	Echo 13	EV 86

Phylogenetic analysis was performed using all the available prototype sequences belonging to enterovirus species B.

Phylogenetic tree is based on complete nucleotide sequence of the VP2 and VP4 genes; GenBank numbers for the prototype sequences used were D00627_CVA-9; M16560_CVB1; AF085363_CVB2; M16572_CVB3; X05690_CVB4; AF114383_CVB5; AF105342_CVB6; AF029859_E1; AY302545_E2; AY302553_E3; AY302557_E4; AF083069_E5; AY302558_E6; AY302559_E7; X84981_E9; X80059_E11; X79047_E12; AY302539_E13; AY302540_E14; AY302541_E15; AY302542_E16; AY302543_E17; AF317694_E18; AY302544_E19; AY302546_E20; AY302547_E21; AY302548_E24; AY302549_E25; AY302550_E26; AY302551_E27; AY302552_E29; AF162711_E30; AY302554_E31; AY302555_E32; AY302556_E33; AY302560_EV69; AF241359_EV73; AY556057_EV74; AY556070_EV75; AJ493062_EV77; AY843297_EV79; AY843298_EV80; AY843299_EV81; AY843300_EV82; AY843301_EV83; DQ902712_EV84; AY843303_EV85; AY843304_EV86; AY843305_EV87; AY843306_EV88; AY843307_EV97; NC_013115.1_EV107; DQ902713_EV100; AY843308_EV101. The GenBank numbers for the in-house sequenced samples from 2003 and 2010 are JQ837913 and JQ837914.

## Discussion

Recombination between enteroviruses is quite frequent, and occurs typically within the same species [1,27]. It was initially thought that the 5’UTR and P1 regions move independently during recombination, and the frequency of recombination was higher in the non-structural coding region [22]. This lead to the conclusion that serotyping by molecular methods involving sequencing of the P1 region should be equivalent to sequencing segments of this region (VP1,2,3 or 4). Several studies have confirmed the finding that sequencing of the VP1 or VP2 regions yields similar typing results compared to sequencing the entire P1 region [1,14,28]. Typing using the VP4 region has yielded conflicting results including studies reporting both consistent and inconsistent results with classical serotyping [28,29]; Perera et al. have noted that the VP4 sequence is unreliable for serotyping of viruses in species B and C [29]. This inconsistency seems to result from recombination events involving the VP4 region [22]. Intuitively, one may still continue to regard the segment coding for the external domains of the capsid to recombine as a block since the VP4 domain remains completely inside the capsid and is inaccessible to antibodies [1].

The amplicon used for serotype determination includes a segment from the 5’UTR, the VP4 region and a segment of the VP2 region; but given the possibility of recombinations outside the VP2-VP3-VP1 region, only the sequence within VP2 was used for the typing of samples in this study. It was noted that if serotype assignment is made based on Blast comparisons with the GenBank database sequences, there is a potential for inaccurate serotype assignment since contemporary isolates submitted into GenBank may have been serotyped based on the VP4 sequence, which can reassort independently of the VP2-VP3-VP1 segment. In this study, the serotype assignment was achieved by constructing a phylogenetic tree with the partial VP2 sequence obtained by sequencing the amplicon and the corresponding VP2 sequences of the original prototype strains [1]. In spite of the genetic drift evident in the sequences of contemporary viruses, this method provided unambiguous serotype assignment. Instances of recombination within the VP2-VP3-VP1 segment have only been rarely reported. Al-Hello and colleagues reported on an isolate that was identified as echovirus 11 by genetic analysis, but could be neutralized by antisera specific for echovirus 11 or CVA-9. A peptide scan analysis confirmed the presence of epitopes recognized by both antisera, whereas a Simplot analysis failed to reveal a recombination event within the capsid [30]. Should reports of isolates with similar properties become more frequent, the very concept of enterovirus serotype might become untenable. Notwithstanding these considerations, at this point in time it remains valuable to serotype isolates, because the serotype is determined by the neutralizing epitopes, and remains important for the molecular epidemiology of these viruses. In addition, the capsid determines the choice of the cellular receptor, consequently the serotype is an important factor to determine the pathogenicity of an isolate [23]; however, the remainder of the genome also contributes to pathogenicity [24] and complete genome sequencing is required for full characterisation of an isolate.

During the 2010 outbreak, a different serotype distribution was noted among infants (< 1year) and older individuals, this difference in distribution of serotypes between age groups has been noted before [31,32]. The serotypes found among infants included in our study have been typically associated with neonatal disease, for example, Coxsackie B3 and B4, echovirus 9, and EV-71. It was interesting to observe one isolate with the VP2 region most similar to the newly described EV-97 [20]. Among the rest of the population, CVA-9 was clearly the predominant serotype. Phylogenetic analysis of the VP2 region from the CVA-9 isolates showed that all the isolates had the same sequences and therefore originated from the same strain.

Comparison of the capsid regions showed the CVA-9_2003 and CVA-9_2010 isolates to be highly homologous and this was corroborated by Simplot anaysis; consequently it is speculated that the sudden increase in detection of CVA-9 in CSF samples is attributable to an increased number of individuals susceptible to CVA-9 in the population rather than to the emergence of an immune escape mutant in 2010. For an infectious agent to cause an outbreak, it is necessary that the herd immunity of the population drops below a threshold that is determined by the basic reproduction number R_0_[33]. For CVA-9 to re-enter the population of Alberta in 2010, either the new isolate was an immune escape mutant against which the population had no prior immunity, or the herd immunity had declined below the threshold. Because of the very high homology in the capsid sequences between the 2003 and 2010 isolates, it does not seem likely that the 2010 isolate was an immune escape mutant.

A comparison with the sequence of the prototype CVA-9 shows that the two Alberta isolates inherited their VP2-VP3-VP1 segment from the prototype, but the VP4 segment shows sequence divergence. For all the other protein coding segments, phylogenetic trees of the nucleotide sequence were constructed to compare the Alberta strains with the prototype strains within species B, and Simplot analysis were performed. Two important observations can be made: firstly, the two Alberta isolates are highly homologous in most regions, with few changes that can be largely attributed to sequence drift; however, recombination has likely occurred in the 2C, 3A, 3C, and 3D regions. Secondly, the Alberta isolates are very different from the prototype CVA-9 in the VP4 segment (supporting the concept that VP4 can be a site for recombination). The 5’NC, structural and non-structural regions appear to be components of a mosaic genome modified from the prototype CVA-9 by recombination and drifting. Similar observations have been made on other contemporary CVA-9 strains [34], and indeed in other contemporary enteroviruses within species B [22,27]. How much the pathogenicity of these CVA-9 viruses has been modified by the non-structural genes, compared to the prototype strain, is unclear [22].

## Conclusion

In summary the sudden increase in cases of aseptic meningitis in Alberta in 2010 was associated with enterovirus CVA-9. The capsid region was highly homologous to the capsid of a 2003 isolate, suggesting that the infections were not the result of the emergence of an immune escape mutant. We thus speculate that the increase in the number of infections may have resulted from a decline in the level of herd immunity against this virus to a level where the virus was able to penetrate the population. When compared to the prototype strain of CVA-9, the Alberta isolates displayed signs of multiple recombination events in addition to genetic drifting.

## Methods

### Screening for enteroviruses

Specimens submitted to the Provincial Laboratory for Public Health (ProvLab) for enterovirus testing from January 1 to December 31, 2010 were included in this study. A total of 4323 samples from patients ranging in age from one day old to 97 years of age were tested; the most common specimen types tested included CSF (n = 2687, 62.16%), plasma (n = 497, 11.50%), nasopharyngeal and throat swabs (n = 213, 4.93%) and stools (n = 103, 2.40%). Viral RNA was extracted using the easyMAG® automated extractor (BioMérieux, Durham, NC, USA) and the extracted nucleic acid was screened for enteroviruses using a previously published NASBA assay [35].

### Serotyping of enteroviruses

Enteroviruses were serotyped using one-step RT-PCR to amplify the partial 5’untranslated region (293 bp), the VP4 region (207 bp) and partial VP2 region (250 bp) using previously described primers 1-EV/RV and 2-EV/RV from RNA extracted directly from the specimen [36]. Sequencing was performed without the need for nested amplification. Amplification was performed using the One-step RT-PCR kit from Qiagen (Ontario, Canada) using 10 μl of 5X buffer, 10 μl of Q solution, 2 μl of 10 mM dNTPs, 2 μl of enzyme, 0.125 μl of RNaseOUT (Life Technologies, Ontario, Canada), 0.8 μM of primers and 5 μl of template nucleic acid in a total volume of 50 μl. The reverse transcription step was performed at 50°C for 30 mins, followed by enzyme activation at 95°C for 15 mins. The amplification protocol included 45 cycles of denaturation at 95°C for 30 seconds, followed by annealing at 55°C for 1.5 minutes and amplification at 72°C for 60 seconds. A final extension step was performed for 10 minutes at 72°C followed by cooling. Amplified products were sequenced in both directions on the ABI PRISM 3130-Avant Genetic Analyzer (Applied Biosystems (ABI), Foster City, CA).

### Complete genome sequencing of CVA-9

Two enterovirus positive samples from 2003 (CVA-9_2003) and 2010 (CVA-9_2010) with a high viral load as estimated by the Ct value, that exhibited strong growth in primary Rhesus Monkey Kidney cells from Diagnostic Hybrids (Ohio, USA) were used. The near-complete genome of these viruses was amplified by long RT-PCR as previously described [37]. The amplicons were sequenced by genome walking and contig assembly was performed using seqscape v2.5 (ABI). The genomic sequences of the CVA-9_2003 and CVA-9_2010 have been deposited in GenBank under accession numbers JQ837913 and JQ837914, respectively.

### Sequence analysis

Sequences were aligned using ClustalX (Version 1.81, March 2000; ftp://ftp-igbmc.u-strasbg.fr/pub/ClustalX/) and phylogenetic analysis was conducted using Treecon [38]. Distance estimation was performed using the Jukes and Cantor distance correction (1969), topology inference was performed using the neighbour joining method, and bootstraping was done using 1000 replicates and the tree was re-rooted at the internode. Simplot analysis were performed using alignments done with CLustalX and the Simplot for Windows v3.5.1 program [39].

## Abbreviations

CSF: Cerebrospinal fluid; CVA: Coxsackie virus A; CVB: Coxsackie virus B; EV: Enterovirus; UTR: Untranslated region; NASBA: Nucleic acid sequence based amplification; RT-PCR: Reverse-transcription polymerase chain reaction; ABI: Applied biosystems; CVA-9: Coxsackie virus A9

## Competing interests

The authors declare that they have no competing interests.

## Authors’ contributions

EC carried out the molecular studies and participated in the sequence alignment. KP, SW and RT conceived of the study, participated in its design, coordination, and analysis. KP wrote the manuscript, SW and RT edited the manuscript. All authors read and approved the final manuscript.
